# Atomistic Band-Structure Computation for Investigating Coulomb Dephasing and Impurity Scattering Rates of Electrons in Graphene

**DOI:** 10.3390/nano11051194

**Published:** 2021-05-01

**Authors:** Thi-Nga Do, Danhong Huang, Po-Hsin Shih, Hsin Lin, Godfrey Gumbs

**Affiliations:** 1 Department of Physics, National Cheng Kung University, Tainan 701, Taiwan; sofia90vn@gmail.com (T.-N.D.); PHShih@phys.ncku.edu.tw (P.-H.S.); 2 US Air Force Research Laboratory, Space Vehicles Directorate (AFRL/RVSU), Kirtland Air Force Base, Albuquerque, NM 87117, USA; 3 Institute of Physics, Academia Sinica, Taipei 11529, Taiwan; nilnish@gmail.com; 4 Department of Physics and Astronomy, Hunter College of the City University of New York, 695 Park Avenue, New York, NY 10065, USA; ggumbs@hunter.cuny.edu

**Keywords:** graphene, scattering, dephasing, relaxation time, band structure, tight-binding model

## Abstract

In this paper, by introducing a generalized quantum-kinetic model which is coupled self-consistently with Maxwell and Boltzmann transport equations, we elucidate the significance of using input from first-principles band-structure computations for an accurate description of ultra-fast dephasing and scattering dynamics of electrons in graphene. In particular, we start with the tight-binding model (TBM) for calculating band structures of solid covalent crystals based on localized Wannier orbital functions, where the employed hopping integrals in TBM have been parameterized for various covalent bonds. After that, the general TBM formalism has been applied to graphene to obtain both band structures and wave functions of electrons beyond the regime of effective low-energy theory. As a specific example, these calculated eigenvalues and eigen vectors have been further utilized to compute the Bloch-function form factors and intrinsic Coulomb diagonal-dephasing rates for induced optical coherence of electron-hole pairs in spectral and polarization functions, as well as the energy-relaxation time from extrinsic impurity scattering of electrons for non-equilibrium occupation in band transport.

## 1. Introduction

**Figure 1:**
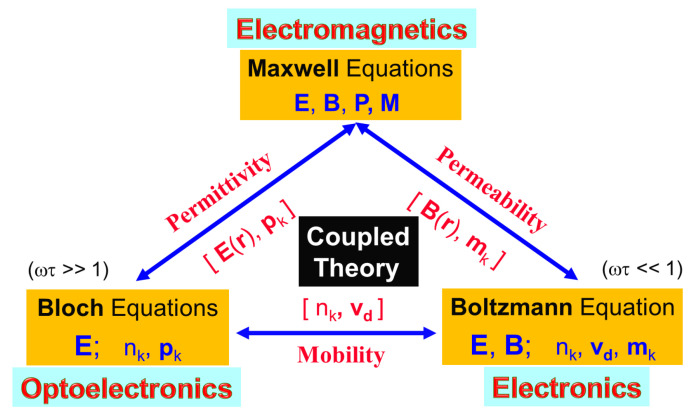
Illustration of a **Device Modeling & Simulation Triangle** for strong-coupling model applied to multi-functional electro-optical devices, where the device electromagnetic, opto-electronic and electronic characteristics are fully described by coupled Bloch, Maxwell, and Boltzmann equations all together.

Very recently, a generalized parameter-free quantum-kinetic model [1,2] based on many-body theory [3,4] has been developed, which is self-consistently coupled with Maxwell equations [5] for an interacting electromagnetic field and with Boltzmann transport equation [6] for a conduction current, as illustrated in Figure 1. Here, being an off-diagonal element in a density matrix, the induced quantum coherence for electron-hole pairs leads to a macroscopic optical polarization field [1] included in the Maxwell equations. Meanwhile, the modified electric field determined from the Maxwell equations can also change the microscopic quantum coherence [1] of electron-hole pairs. In this way, a self-consistent loop is constructed between electrons in the quantum-kinetic model and electric field in the Maxwell equations. This theory aims at enabling first-principles computations of ultra-fast dynamics for non-thermal photo-generated electron-hole pairs in undoped semiconductors [1,2]. At the same time, this a theory is also able to simultaneously describe electromagnetic, optical and electrical properties of crystal materials and their interplay all together. More importantly, the numerical output of this first-principles dynamics model can be utilized as an input for material optical and transport properties to be fed into a next-stage simulation software facilitated by finite-element methods, such as COMSOL Multiphysics [7], for devices with various configurations. Consequently, device characteristics can be accurately predicted beyond the linear-response regime [3,4] for numerical bottom-up design and engineering. However, such a quantum-kinetic model itself requires an input from wave functions and band structures associated with different host materials in devices.

In Figure 1, we introduce the product of field frequency (ω) with the carrier momentum-relaxation time ($\tau_{p}$). The situations with $\omega \tau_{p}\gg 1$ and $\omega \tau_{p}\ll 1$ correspond separately to optical and bias field regimes, while $\omega \tau_{p}\approx 1$ uniquely specifies the terahertz regime with dual optical and bias field characteristics. The bridging connection between the Maxwell [5] and semiconductor Bloch [8,9] equations is provided by the induced optical-polarization field P(r,t) as a quantum-statistical average of the electric-dipole moment with the induced microscopic optical coherence $p_{j}\left(k,t\right)$ with *j* the band index. The bridging connection between the Maxwell [5] and Boltzmann transport [6] equations, on the other hand, is fulfilled by the optically-induced magnetization field M(r,t) as a quantum-statistical average of the induced microscopic magnetic-dipole moment $m_{j}\left(k,t\right)$ from spins or orbital angular momentum. Finally, the bridging connection between the semiconductor Bloch [8,9] and Boltzmann transport [6] equations is facilitated by the bias-induced macroscopic center-of-mass drift velocity $v_{d}\left(t\right)$ as a non-equilibrium quantum-statistical average of the microscopic electron group velocities $v_{j}\left(k\right)$ from multi-band dispersions for modifying optical-transition properties of driven carriers within the center-of-mass frame due to relative scattering motions of carriers.

The first-principles computation of electron Bloch wave function and band dispersion of a targeted material can be performed by employing the well-known Kohn-Sham density-functional theory [10]. Meanwhile, the tight-binding model [11,12,13,14,15] for solid crystals is usually considered as an alternative approach for computing electronic band structure using an approximate set of orbital wave functions based upon superposition of bond-orbital states for isolated atoms sitting at different lattice sites. In fact, this method is closely related to the linear combination of atomic orbitals method [16] adopted commonly in quantum chemistry. Such a real-space tight-binding model can be applied to a lot of solids, even including a magnetic field, Ref. [17] and it is proved giving rise to good qualitative results [18]. Moreover, this method can be combined with other models to produce better results whenever the tight-binding model fails. Here, we would like to emphasize that although the tight-binding model is only a one-electron model in nature, it indeed provides a basis for more advanced computations [11], such as the computation of surface states, application to various kinds of many-body problems, and quasi-particle calculation [19].

Historically, the family of carbon-based materials can be characterized into two distinct crystal forms, i.e., the isotropic diamond and anisotropic graphite. Recently, their allotropes, such as fullerenes and carbon nanotubes, entered into play and expanded to graphene, which is a unique material consisting of a two-dimensional lattice of carbon atoms with a honeycomb symmetry. Graphene stands for an physically interesting system [20,21], and becomes very promising for future device applications. On the atomic level, e.g., density-functional theory, electron certainly follows the Schrodinger equation. However, by using an approximate effective-mass Hamiltonian [22,23] for low-energy electrons near the *K* or K′ valley, the quasi-particles are found to satisfy the relativistic Dirac equation for massless fermions. Today, the extensive investigations on various graphene systems have turned into a broad research field for qualitatively new two-dimensional systems [24]. Up to now, the basic properties of novel 2D allotropes of carbon, including graphene [22,23], graphene bilayer [25,26,27], multi-layer graphene [28,29], graphene on a silicon carbide substrate [30], are well known and the basis of graphene physics becomes well established.

In recent years, by using the low-energy Dirac Hamiltonian [4], we have extensively explored varieties of dynamical properties of electrons in graphene and other two-dimensional materials, including Landau quantization [18,31,32,33,34,35], many-body optical effects [36,37,38,39,40,41], band and tunneling transports [42,43,44,45,46,47,48,49,50], etc. In this paper, we particularly focus on the application of computed electronic states and band structures from a tight-binding model to the calculations of Coulomb and impurity scatterings of electrons in graphene on the basis of a many-body theory [3,4], where the former and latter determine the lineshape [1] of an absorption peak and the transport mobility [44], respectively.

The rest of paper is organized as follows. In Section 2, we present a general description of tight-binding model for novel two-dimensional materials. Section 3 is devoted to discuss the Slater-Koster approximation for bonding parameters and bonding integrals. We acquire the parameter values in Section 4 and obtain graphene wave functions and band structures. We study the Coulomb diagonal-dephasing rate of electron-hole pairs in undoped graphene in Section 5, as well as the impurity scattering rate of conduction electrons in Section 6, respectively. Finally, a brief summary is presented in Section 7 along with some remarks.

## 2. General Description of Tight-Binding Model

For completeness, we start with tight-binding model [14] for computing complete band structures of two-dimensional materials. The advantage of tight-binding model is easily incorporating a magnetic field through the so-called Peierls substitution in the phase of a hopping integral [51]. In quantum mechanics, the single-electron static Schrödinger equation is written as [52]

(1) $$\hat{H}\;\Phi_{k}\left(r\right)=E_{k}\;\Phi_{k}\left(r\right)\;,$$

where $\Phi_{k}\left(r\right)$ is the Bloch wave function, $E_{k}$ the eigen-energy, and ***k*** is the wave vector of electrons within the first Brillouin zone of two-dimensional materials. The Hamiltonian operator $\hat{H}$ in Equation (1) takes a general form

(2) $$\hat{H}={\hat{H}}_{0}+V\left(r\right)\;,$$

in which the kinetic-energy operator ${\hat{H}}_{0}$ is

(3) $${\hat{H}}_{0}=-\frac{\hbar^{2}}{2m_{e}}\;\nabla_{r}^{2}$$

with free-electron mass $m_{e}$, while the potential energy V(r) for an electron within the lattice of two-dimensional materials is given by [11]

(4) $$V\left(r\right)=V_{\mathrm{ion}}\left(r\right)+\Delta V_{L}\left(r\right)$$

with $V_{\mathrm{ion}}\left(r\right)$ and $\Delta V_{L}\left(r\right)$ specifying the potentials of a single ion and that for the rest of ions, respectively. The Bloch wave function $\Phi_{k}\left(r\right)$ of electrons in Equation (1) can be decomposed into a linear combination of a set of orbital wave functions $\left\{\phi_{\beta ,k}\left(r\right)\right\}$ within the first Brillouin zone, leading to [11]

(5) $$\Phi_{k}\left(r\right)=\sum_{\beta }\;C_{\beta }\;\phi_{\beta ,k}\left(r\right)\;,$$

where the index β labels all the atomic orbitals of the lattice of two-dimensional materials. The expansion coefficient $C_{\beta }$ introduced in Equation (5) can be decided from

(6) $$C_{\beta }=\int d^{3}r\;\phi_{\beta ,k}^{*}\left(r\right)\;\Phi_{k}\left(r\right)\;,$$

where the orthonormal property for the set of orbital wave functions $\left\{\phi_{\beta ,k}\left(r\right)\right\}$ has been adopted.

Applying the method of linear combination of atomic orbitals (LCAO) of all ions on the lattice [16], we further express each orbital wave function $\phi_{\beta ,k}\left(r\right)$ in Equation (5) by a linear combination of bond-orbital states $\left\{\psi_{\beta }\left(r-R_{j}\right)\right\}$ within a unit cell in real space, namely

(7) $$\phi_{\beta ,k}\left(r\right)=\frac{1}{\sqrt{N}}\sum_{j=1}^{N}\;\exp\left(ik\cdot R_{j}\right)\;\psi_{\beta }\left(r-R_{j}\right)\;,$$

where *j* is the index for all bonded lattice ions, $R_{j}$ the lattice-ion position vector, and *N* the total number of atoms within the unit cell. Here,

(8) $$\psi_{\beta }\left(r-R_{j}\right)=\frac{1}{\sqrt{N}}\sum_{k}\;\exp\left(-ik\cdot R_{j}\right)\;\phi_{\beta ,k}\left(r\right)$$

is termed as the localized Wannier function for the β orbital of a bonded lattice ion at the site $R_{j}$, which satisfies the single-ion Schrödinger equation [16]

(9) $$\left[{\hat{H}}_{0}+V_{\mathrm{ion}}\left(r\right)\right]\psi_{\beta }\left(r-R_{j}\right)=\varepsilon_{j,\beta }\;\psi_{\beta }\left(r-R_{j}\right)$$

with $\varepsilon_{j,\beta }$ being the βth energy levels of electrons within an ion at the lattice site $R_{j}$.

Combining results in Equations (5) and (7), we acquire the following full LCAO expansion of a Bloch wave function [11]

(10) $$\Phi_{k}\left(r\right)=\frac{1}{\sqrt{N}}\sum_{j=1}^{N}\;\sum_{\beta }\;C_{\beta ;\;j}\left(k\right)\;\psi_{\beta }\left(r-R_{j}\right)$$

with $C_{\beta ;\;j}\left(k\right)=C_{\beta }\;\exp\left(ik\cdot R_{j}\right)$. At the same time, using Equation (5), we find from Equation (1) that

(11) $$\sum_{\beta }\;C_{\beta }\;\int d^{2}r\;\phi_{\alpha ,k}^{*}\left(r\right)\;\hat{H}\;\phi_{\beta ,k}\left(r\right)=E\left(k\right)\;\sum_{\beta }\;C_{\beta }\;\int d^{2}r\;\phi_{\alpha ,k}^{*}\left(r\right)\;\phi_{\beta ,k}\left(r\right)\;,$$

or equivalently, the following eigenvalue equation

(12) $$\sum_{\beta }\;H_{\alpha ,\beta }\left(k\right)\;C_{\beta }=E\left(k\right)\;\sum_{\beta }\;\delta_{\alpha ,\beta }\;C_{\beta }=E\left(k\right)\;C_{\alpha }\;.$$

As a result, the eigenvalue $E_{n}\left(k\right)$ can be determined from the secular determinant of Equation (12) for any given ***k***, yielding

(13) $$Det\left\{\;H_{\alpha ,\beta }\left(k\right)-E_{n}\left(k\right)\;\delta_{\alpha ,\beta }\;\right\}=0\;,$$

and the orthonormal-eigenvectors $\left\{C_{n,\beta }\right\}$ are also obtained, corresponding to the eigenvalue $E_{n}\left(k\right)$ at given ***k***, where the index *n* labels different quantized energy bands of two-dimensional materials. Explicitly, using Equation (7), we obtain the Hamiltonian matrix elements in Equation (12) as [11]

(14) $$H_{\alpha \beta }\left(k\right)=\frac{1}{N}\sum_{i,j=1}^{N}\;\exp\left(ik\cdot R_{j}\right)\;H_{i\alpha ,\;j\beta }\;,$$

in which $R_{ij}=R_{j}-R_{i}$, and

(15) $$H_{i\alpha ,\;j\beta }=\int d^{2}r\;\psi_{\alpha }^{*}\left(r-R_{i}\right)\;\left[{\hat{H}}_{0}+V_{\mathrm{ion}}\left(r\right)\right]\;\psi_{\beta }\left(r-R_{j}\right)+\int d^{2}r\;\psi_{\alpha }^{*}\left(r-R_{i}\right)\;\Delta V_{L}\left(r\right)\;\psi_{\beta }\left(r-R_{j}\right)\;.$$

In fact, we know from Equation (9) that

(16) $$\int d^{2}r\;\psi_{\alpha }^{*}\left(r-R_{i}\right)\;\left[{\hat{H}}_{0}+V_{\mathrm{ion}}\left(r\right)\right]\;\psi_{\beta }\left(r-R_{j}\right)=\varepsilon_{j,\beta }\;\delta_{\alpha ,\beta }\;\delta_{i,j}\;,$$

(17) $$\int d^{2}r\;\psi_{\alpha }^{*}\left(r-R_{i}\right)\;\Delta V_{L}\left(r\right)\;\psi_{\beta }\left(r-R_{j}\right)\equiv \left\{\begin{array}{cc} C_{\Sigma }\;\delta_{\alpha ,\beta } & \mathrm{if}\;i=j\;,\\ t_{\alpha \beta }\left(R_{ij}\right) & \mathrm{if}\;i\neq j\;, \end{array}\right.$$

where $\left(\varepsilon_{j,\beta }+C_{\Sigma }\right)$ represents the site energy, and $t_{\alpha \beta }\left(R_{ij}\right)$ is usually called the two-center (or hopping) integral [14].

As a final step, with the help from Equation (10), we arrive at the full expression for Hamiltonian matrix elements, given by

(18) $$\begin{array}{l} \int d^{2}r\;\Phi_{n^{\prime },k^{\prime }}^{*}\left(r\right)\;\hat{H}\;\Phi_{n,k}\left(r\right)=\frac{1}{N}\sum_{j,j^{\prime }=1}^{N}\;\sum_{\alpha ,\beta }\;C_{n^{\prime },\alpha }^{*}\;C_{n,\beta }\;\exp\left(ik\cdot R_{j}-ik^{\prime }\cdot R_{j^{\prime }}\right)\;H_{j^{\prime }\alpha ,\;j\beta }\\ =\frac{\left(\varepsilon_{j,\beta }+C_{\Sigma }\right)}{N}\;\sum_{j=1}^{N}\;\sum_{\beta }\;C_{n^{\prime },\beta }^{*}\;C_{n,\beta }\;\exp\left[i\left(k-k^{\prime }\right)\cdot R_{j}\right]\\ +\frac{1}{N}\sum_{j,j^{\prime }=1}^{N}{\;}^{\prime }\;\sum_{\alpha ,\beta }\;C_{n^{\prime },\alpha }^{*}\;C_{n,\beta }\;\exp\left(ik\cdot R_{j}-ik^{\prime }\cdot R_{j^{\prime }}\right)\;t_{\alpha \beta }\left(R_{j^{\prime }j}\right)\;, \end{array}$$

where the primed summation in the second term of the right-hand side of the last equation excludes the contribution from $j=j^{\prime }$, and $C_{n,\beta }$ can be obtained from the calculated eigenvector from Equation (12). The matrix elements for other physical operators can be computed in a similar way.

## 3. Slater-Koster Approximation for Hopping Integrals

To seek for the feasibility of fast numerical computation, we introduce a parameterized process for the tight-binding model described in Section 2. For the Coulomb interaction between electron and ion within an atom, the potential field presents a spherical symmetry. Therefore, the energy levels labeled by the radial quantum number n=1,2,⋯ will degenerate with the angular-momentum quantum number ℓ=0,1,⋯,n−1, as well as the magnetic quantum number m=−ℓ,⋯,0,⋯,ℓ [52]. Consequently, there exists a total orbital degeneracy $n^{2}$ (excluding the spin-degeneracy). Customarily, we specify these orbitals by ℓ=0,1,2,3,⋯ for {s,p,d,f,⋯} orbitals.

**Figure 2:**
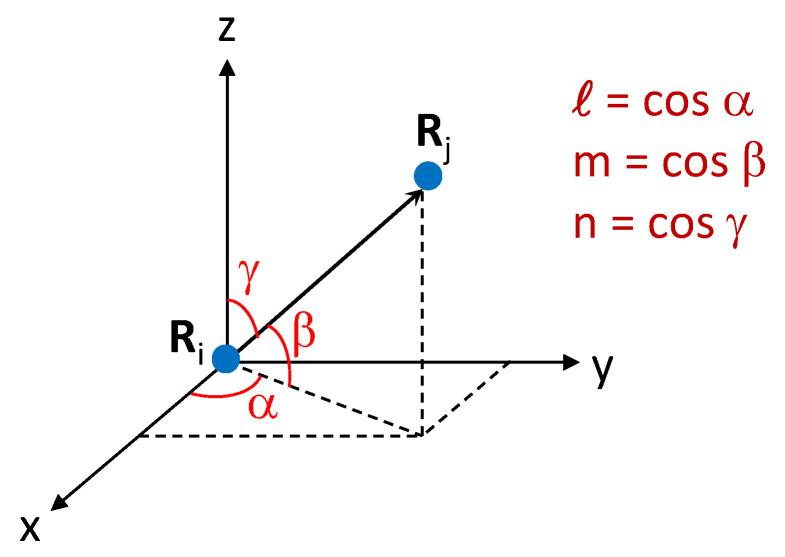
Illustration for three directional cosines ℓ,m,n in a three-dimensional position space for two atoms sitting at $r=R_{i}$ and $r=R_{j}$, respectively.

In order to describe the chemical bonds between a pair of atoms inside a lattice, we often adopt the concept of overlapping electronic orbitals {s,p,d,f,⋯}. To further specify the spatial direction of the chemical bonding between two atoms at the lattice sites $R_{i}$ and $R_{j}$, we have to rely on three directional cosines ℓ,m,n, as defined in Figure 2.

**Figure 3:**
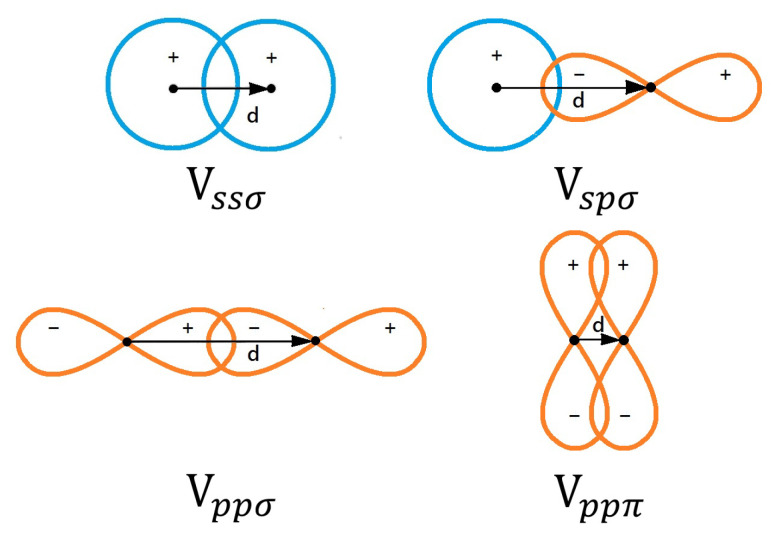
Illustrations for π and σ bonding of atomic *s* and *p* orbitals. Details on description of these bonding orbitals in this figure can be found in Ref. [11].

**Figure 4:**
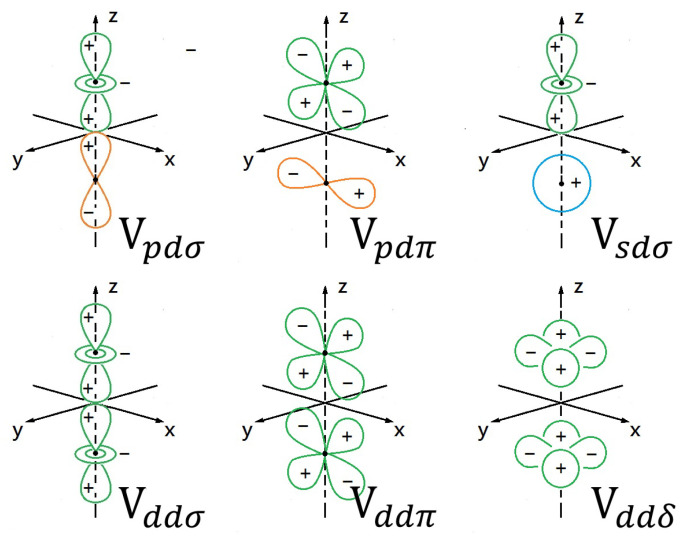
Illustrations for π, σ and δ bonding of atomic *s*, *p*, and *d* orbitals. Details on description of these bonding orbitals in this figure can be found in Ref. [11].

Considering *s* and *p* orbitals as an example, we display their possible bonding potentials $V_{\ell ,\ell^{\prime };\sigma \left(\pi \right)}$ in Figure 3 for *s*, *p* orbitals and four different configurations, including σ and π bonds. Meanwhile, we also list six different π, σ, δ bonding configurations in Figure 4 for *s*, *p*, *d* orbitals.

To speed up numerical computations, the bonding potentials $V_{\ell ,\ell^{\prime };\sigma \left(\pi \right)}$ for $\ell ,\;\ell^{\prime }=s,\;p,\;d$ in Figure 3 and Figure 4 are usually parameterized as: [53] $V_{\ell ,\ell^{\prime };\xi }=\left(\hbar^{2}/m_{e}d^{2}\right)\;\eta_{\ell \ell^{\prime };\xi }$, $V_{\ell ,d;\xi }=\left(\hbar^{2}r_{d}^{3/2}/m_{e}d^{7/2}\right)\;\eta_{\ell ,d;\xi }$ and $V_{d,d;\xi }=\left(\hbar^{2}r_{d}^{3}/m_{e}d^{5}\right)\;\eta_{d,d;\xi }$, where *d* and $r_{d}$ represent the bonding length and atomic radius, and ξ=σ,π,δ are for various bond configurations. Here, the dimensionless bonding parameters $\eta_{\ell ,\ell^{\prime };\xi }$ for different bonding types are listed in Table 1.

By using these parameterized bonding potentials $V_{\ell ,\ell^{\prime };\xi }$, $V_{\ell ,d;\xi }$ and $V_{d,d;\xi }$, we are able to compute further the hopping integrals $t_{\alpha \beta }\left(R_{ij}\right)$ based on the Slater-Koster approximation [14], and some commonly-used results are shown in Table 2.

## 4. Tight-Binding Model for Graphene Band Structure

To seek for an application, we use the general theory, as developed in Section 2 and Section 3, for novel two-dimensional graphene material in order to obtain its electronic wave functions and band structures for the full first Brillouin zone [54]. In this way, we are able to study scattering dynamics with respect to high-energy electrons in graphene resulted from Coulomb interactions between either pair of electrons or between electrons and ionized impurity atoms.

**Figure 5:**
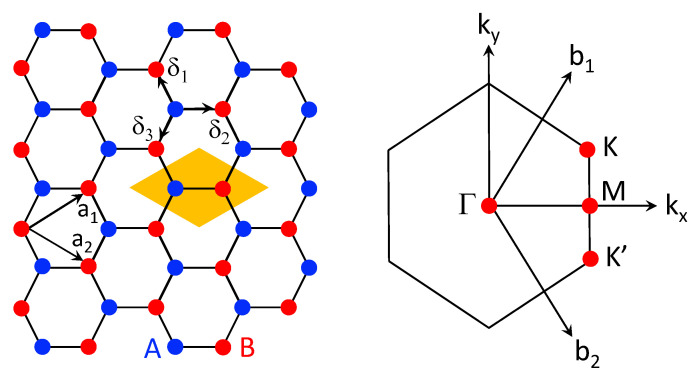
(**Left**) A diagram illustrating the hexagonal-lattice structure of a monolayer graphene with two sublattices *A* (blue) and *B* (red) with the Bravis lattice vectors $a_{1,2}$ and the nearest-neighbor lattice vectors $\delta_{1,\;2,\;3}$. (**Right**) the first Brillouin zone of graphene with labeled high-symmetry points $\Gamma ,\;M,\;K,\;K^{\prime }$ in the k–space with reciprocal-lattice vectors $b_{1,2}$. In the left panel, a unit cell is shown in the shaded region in yellow.

Monolayer graphene displays a hexagonal (or honeycomb) lattice structure of carbon atoms, as illustrated in Figure 5, where each carbon atom is connected by σ covalent bonds with its three nearest neighbors. The electronic orbitals of a carbon atom are characterized as $1s^{2}\;2s^{2}\;2p^{2}$. However, the unique energy difference between the 2s and 2p orbitals favors the appearance of a mixed state of these two orbitals. The first-principles density-functional calculations reveal that it becomes energetically favorable to move an electron from the 2s orbital to the 2p orbital in this mixed state. Since the 2p orbitals include $2p_{x},\;2p_{y},\;2p_{z}$, as a result, each of these three 2p orbitals will accommodate one electron, leading to the *x*–*y* orbitals within the plane of the lattice, as well as the *z* orbital out of the lattice plane. Here, two electrons in the mixed *x*–*y* orbitals form the higher-energy σ bonds, while the remaining electron in the *z* orbital leads to the lower-energy π bonds, i.e., a side-on overlap of the 2p-orbital wave functions. Consequently, these π-bond electrons give rise to the low-energy bands of graphene and will be studied exclusively based on a tight-binding model.

From Equation (7), we know the wave function for π-bond ($p_{z}$-orbital) electrons in graphene can be expressed as

(19) $$\phi_{p_{z},k}^{A,B}\left(r\right)=\frac{1}{\sqrt{N}}\sum_{j=1}^{N}\;\exp\left(ik\cdot R_{j}\right)\;\psi_{p_{z}}^{A,B}\left(r-R_{j}\right)\;,$$

where $k\equiv (k_{x},\;k_{y})$, $R_{j}\equiv \left(R_{j}^{x},\;R_{j}^{y}\right)=m_{j}a_{1}+n_{j}a_{2}$ represents the Bravis lattice-site vectors as indicated in Figure 5, and indexes A,B refer to two sublattices of graphene. By including both sublattices *A* and *B*, we have

(20) $$\phi_{p_{z},k}\left(r\right)=a_{k}\;\phi_{p_{z},k}^{A}\left(r\right)+b_{k}\;\phi_{p_{z},k}^{B}\left(r\right)\;,$$

where $a_{k}$ and $b_{k}$ are two elements of the eigenvector corresponding to the eigenvalue equation with respect to two sublattices. Specifically, from Equations (11) and (20), we arrive at the matrix-form Schrödinger equation

(21) $$\left[\begin{array}{cc} H_{AA}\left(k\right) & H_{AB}\left(k\right)\\ H_{BA}\left(k\right) & H_{BB}\left(k\right) \end{array}\right]\left[\begin{array}{c} a_{k}\\ b_{k} \end{array}\right]=E_{n}\left(k\right)\left[\begin{array}{cc} S_{AA}\left(k\right) & S_{AB}\left(k\right)\\ S_{BA}\left(k\right) & S_{BB}\left(k\right) \end{array}\right]\left[\begin{array}{c} a_{k}\\ b_{k} \end{array}\right]\;,$$

where $S_{\ell \ell^{\prime }}\left(k\right)=\langle \;\phi_{p_{z},k}^{\ell }\;|\;\phi_{p_{z},k}^{\ell^{\prime }}\;\rangle$, $H_{\ell \ell^{\prime }}\left(k\right)=\langle \;\phi_{p_{z},k}^{\ell }\;|\;\hat{H}\;|\;\phi_{p_{z},k}^{\ell^{\prime }}\;\rangle$, $\ell ,\;\ell^{\prime }=$ *A* or *B*, and $E_{n}\left(k\right)$ represents the eigen-energies of π-bond electrons with n=1,2 labeling two graphene low-energy bands determined by the secular determinant: $Det{\left\{\;H_{\ell \ell^{\prime }}\left(k\right)-E_{n}\left(k\right)\;S_{\ell \ell^{\prime }}\left(k\right)\;\right\}}_{2\times 2}=0$.

As in Equation (7), we can rewrite the orbital wave function $\phi_{p_{z},k}\left(r\right)$ in Equation (20) approximately only by its near-neighbor decomposition, yielding

(22) $$\phi_{p_{z},k}\left(r\right)=\frac{1}{\sqrt{N_{c}}}\sum_{\ell \in A,B}\;\exp\left(ik\cdot R_{\ell }\right)\sum_{j=1}^{N_{c}}\;a_{jk}\;\psi_{p_{z}}\left(r-R_{\ell }+\Delta_{j}\right)\;,$$

and then, the eigenvalue equation turns into $Det{\left\{\;H_{jj^{\prime }}\left(k\right)-E_{s}\left(k\right)\;S_{jj^{\prime }}\left(k\right)\;\right\}}_{N_{c}\times N_{c}}=0$ with eigen-vectors ${\left\{a_{jk}\right\}}_{N_{c}\times 1}$, where $N_{c}$ represents the number of near-neighbor atoms within a unit cell, $\Delta_{j}$ stands for the lattice vectors of the near-neighbor atoms relative to the sublattice site $R_{\ell }$, and $a_{jk}=a_{k}\exp\left(-ik\cdot \Delta_{j}\right)$. Moreover, we find $H_{jj^{\prime }}\left(k\right)=\varepsilon_{2}\;S_{jj^{\prime }}\left(k\right)+t_{jj^{\prime }}\left(k\right)$, where $\varepsilon_{2}$ stands for the second energy level of electrons within a carbon atom,

(23) $$S_{jj^{\prime }}\left(k\right)=\sum_{\ell \in A,B}\;\exp\left(ik\cdot R_{\ell }\right)\int d^{2}r\;\psi_{p_{z}}^{*}\left(r-R_{\ell }+\Delta_{j}\right)\;\psi_{p_{z}}\left(r-R_{\ell }+\Delta_{j^{\prime }}\right)$$

is the overlap integral, while

(24) $$t_{jj^{\prime }}\left(k\right)=\sum_{\ell \in A,B}\;\exp\left(ik\cdot R_{\ell }\right)\int d^{2}r\;\psi_{p_{z}}^{*}\left(r-R_{\ell }+\Delta_{j}\right)\;\Delta V_{L}\left(r\right)\;\psi_{p_{z}}\left(r-R_{\ell }+\Delta_{j^{\prime }}\right)$$

is the hopping integral.

For simplicity, we would omit the orbital index $p_{z}$ from now on. Without loss of generality, we can assume that the vectors that connect sublattice *A* site to the equivalent site on the *B* sublattice is $\delta_{3}$, as seen in Figure 5. As a result, the hopping and overlap amplitudes between the nearest neighbor (nn) and the next-nearest neighbor (nnn) can be computed explicitly from Equations (23) and (24), leading to

(25) $$\begin{array}{ccc} t_{AB}\left(k\right) & = & t_{BA}^{*}\left(k\right)=\gamma^{*}\left(k\right)\;t_{nn}\;,\\ t_{AA}\left(k\right)-C_{p}S_{AA} & = & t_{BB}\left(k\right)-C_{p}S_{BB}=2\;t_{nnn}\sum_{i=1}^{3}\;\cos\left(k\cdot a_{i}\right)=(|\gamma \left(k\right){|}^{2}-3)\;t_{nnn}\;,\\ S_{AB}\left(k\right) & = & S_{BA}^{*}\left(k\right)=\gamma^{*}\left(k\right)\;s_{nn}\;,\\ S_{AA}\left(k\right) & = & S_{BB}\left(k\right)=1+{\left(|\gamma \left(k\right)\right|}^{2}-3)\;s_{nnn}\approx 1\;, \end{array}$$

where $a_{3}\equiv a_{1}-a_{2}$, $\gamma \left(k\right)=1+\exp\left(ik\cdot a_{1}\right)+\exp\left(ik\cdot a_{2}\right)$, and the hopping and overlap integrals are calculated as

(26) $$\begin{array}{ccc} C_{p} & = & \int d^{2}r\;\psi_{A}^{*}\left(r\right)\;\Delta V_{L}\left(r\right)\;\psi_{A}\left(r\right)=\int d^{2}r\;\psi_{B}^{*}\left(r\right)\;\Delta V_{L}\left(r\right)\;\psi_{B}\left(r\right)\;,\\ t_{nn} & = & \int d^{2}r\;\psi_{A}^{*}\left(r\right)\;\Delta V_{L}\left(r\right)\;\psi_{B}\left(r+\delta_{3}\right)\;,\\ t_{nnn} & = & \int d^{2}r\;\psi_{A}^{*}\left(r\right)\;\Delta V_{L}\left(r\right)\;\psi_{A}\left(r+a_{1}\right)=\int d^{2}r\;\psi_{B}^{*}\left(r\right)\;\Delta V_{L}\left(r\right)\;\psi_{B}\left(r+a_{1}\right)\;,\\ s_{nn} & = & \int d^{2}r\;\psi_{A}^{*}\left(r\right)\;\psi_{B}\left(r+\delta_{3}\right)\;,\\ s_{nnn} & = & \int d^{2}r\;\psi_{A}^{*}\left(r\right)\;\psi_{A}\left(r+a_{1}\right)=\int d^{2}r\;\psi_{B}^{*}\left(r\right)\;\psi_{B}\left(r+a_{1}\right)\;. \end{array}$$

Particularly, the results for these tight-binding model parameters in Equation (26) for band structures are presented in Table 3, which have been computed from listed bonding parameters in Table 1 and bonding integrals in Table 2.

Finally, from the eigenvalue equation $Det{\left\{\;t_{\ell \ell^{\prime }}\left(k\right)-E\left(k\right)\;S_{\ell \ell^{\prime }}\left(k\right)\;\right\}}_{2\times 2}=0$ in Equation (21) for $\ell ,\;\ell^{\prime }=A,\;B$, we obtain an explicit expression

(27) $$E^{2}\left(k\right)\;Det\left\{\overset{↔}{S}\right\}-E\left(k\right)\left[S_{AA}t_{BB}+S_{BB}t_{AA}-S_{AB}t_{BA}-S_{BA}t_{AB}\right]+Det\left\{t\right\}=0\;,$$

where, by setting $s_{nnn}=0$, we have three coefficients

(28) $$\begin{array}{ccc} & & Det\left\{\overset{↔}{S}\right\}=1-s_{nn}^{2}\;{\left|\gamma \left(k\right)\right|}^{2}\;,\\ & & Det\left\{\overset{↔}{t}\right\}=(|\gamma \left(k\right){|}^{2}-{3|)}^{2}\;t_{nnn}^{2}-t_{nn}^{2}\;{\left|\gamma \left(k\right)\right|}^{2}\;,\\ & & S_{AA}t_{BB}+S_{BB}t_{AA}-S_{AB}t_{BA}-S_{BA}t_{AB}=2\left[{\left(|\gamma \left(k\right)\right|}^{2}-3)\;t_{nnn}-t_{nn}s_{nn}\;{\left|\gamma \left(k\right)\right|}^{2}\right]\;. \end{array}$$

This leads to the explicit solution of Equation (27), namely [55]

(29) $$E_{\lambda }\left(k\right)=\left(\varepsilon_{2}+C_{p}\right)+\frac{{\left(|\gamma \left(k\right)\right|}^{2}-3|)\;t_{nnn}-t_{nn}s_{nn}\;{\left|\gamma \left(k\right)\right|}^{2}+\lambda \sqrt{D(k)}}{1-s_{nn}^{2}{\left|\gamma \left(k\right)\right|}^{2}}\;,$$

where λ=±1 correspond to valence (−1) and conduction (+1) bands, respectively, and

(30) $$\begin{array}{ccc} D(k) & = & {\left[{\left(|\gamma \left(k\right)\right|}^{2}-3)\;t_{nnn}-t_{nn}s_{nn}\;{\left|\gamma \left(k\right)\right|}^{2}\right]}^{2}-(1-s_{nn}^{2}{\;|\gamma \left(k\right)|}^{2})\\ & \times & \left[{\left(|\gamma \left(k\right)\right|}^{2}-{3|)}^{2}\;t_{nnn}^{2}-t_{nn}^{2}\;{\left|\gamma \left(k\right)\right|}^{2}\right]={\left|\gamma \left(k\right)\right|}^{2}{\left[{\left(|\gamma \left(k\right)\right|}^{2}-3)\;t_{nnn}\;s_{nn}+t_{nn}\right]}^{2}\;. \end{array}$$

By using the result in Equations (29) and (30) can be rewritten as

(31) $$\begin{array}{ccc} E_{\lambda }\left(k\right) & = & \left(\varepsilon_{2}+C_{p}\right)+\frac{{\left(|\gamma \left(k\right)\right|}^{2}-3)\;t_{nnn}[1+\lambda |\gamma \left(k\right)|\;s_{nn}]-t_{nn}s_{nn}{\left|\gamma \left(k\right)\right|}^{2}+\lambda \;\left|\gamma \left(k\right)\right|\;t_{nn}}{1-s_{nn}^{2}{\left|\gamma \left(k\right)\right|}^{2}}\\ & \approx & \left(\varepsilon_{2}+C_{p}\right)+\frac{{\left(|\gamma \left(k\right)\right|}^{2}-3)\;t_{nnn}-t_{nn}s_{nn}{\left|\gamma \left(k\right)\right|}^{2}+\lambda \;t_{nn}\;\left|\gamma \left(k\right)\right|}{1-s_{nn}^{2}{\left|\gamma \left(k\right)\right|}^{2}}\;. \end{array}$$

**Figure 6:**
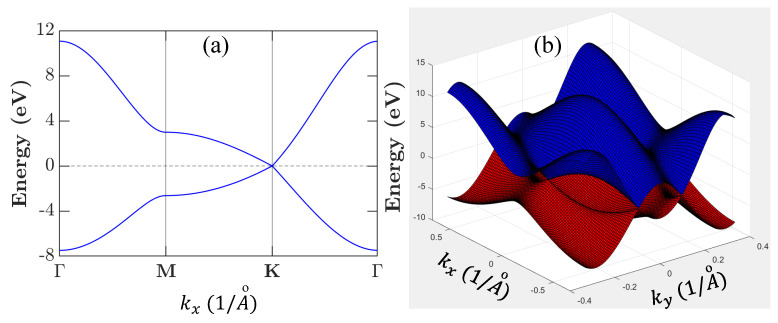
Calculated dispersion of energy bands for graphene. Panel (**a**) displays 2D plot for energy dispersion of graphene electrons. Panel (**b**) shows 3D plot for upper and lower bands touched at six Dirac points (three ***K*** and three $K^{\prime }$ valleys), at which the energy is set to be zero.

By setting $C_{p}+\varepsilon_{2}=0$ as the reference point for energy, the result in Equation (31) is plotted in Figure 6 by employing the graphene structural parameters listed in Table 3.

Furthermore, by using the result in Equation (31), two elements of the eigenvector, $a_{k}^{\lambda }$ and $b_{k}^{\lambda }$, are found to be

(32) $$\begin{array}{ccc} a_{k}^{\lambda } & = & \left\{\frac{\gamma^{*}\left(k\right)\;\left[E_{\lambda }\left(k\right)\;s_{nn}-t_{nn}\right]}{{\left(|\gamma \left(k\right)\right|}^{2}-3)\;t_{nnn}-E_{\lambda }\left(k\right)}\right\}b_{k}^{\lambda }\;,\\ b_{k}^{\lambda } & = & \frac{{\left|(|\gamma \left(k\right)\right|}^{2}-3)\;t_{nnn}-E_{\lambda }\left(k\right)|}{\sqrt{|\gamma^{*}\left(k\right)\;\left[t_{nn}-E_{\lambda }\left(k\right)\;s_{nn}\right]{|}^{2}+{\left|(|\gamma \left(k\right)\right|}^{2}-3{)\;t_{nnn}-E_{\lambda }\left(k\right)|}^{2}}}\;. \end{array}$$

As known experimentally, both the nearest-neighbor (nn) overlap and the next-nearest-neighbor (nnn) hopping integrals are much smaller than the nearest-neighbor (nn) hopping integral. By neglecting some constants, the dispersion in Equation (31) can be further simplified as

(33) $$E_{\lambda }\left(k\right)\approx 2\;t_{nnn}^{\prime }\sum_{i=1}^{3}\;\cos\left(k\cdot a_{i}\right)+\lambda \;t_{nn}{\left[3+2\sum_{i=1}^{3}\;\cos\left(k\cdot a_{i}\right)\right]}^{1/2}\;,$$

where $t_{nnn}^{\prime }=t_{nnn}-s_{nn}t_{nn}$ is the corrected hopping amplitude.

## 5. Coulomb Diagonal-Dephasing Rate for Optical Coherence in Undoped Graphene

The quantum coherence of electrons is associated with the off-diagonal elements of their density matrix. The presence of an external field can induce coherence between two quantum states of electrons if the field frequency matches the energy separation between the two relevant electronic states. Dephasing refers to a physics mechanism which recovers classical behavior from a quantum system, and it quantifies the time required for electrons to lose their field-induced quantum coherence. Diagonal-dephasing rate connects to the ways in which coherence caused by perturbation decays over time, and then, the system goes back to the state before perturbation [1]. This is an important effect in molecular and atomic spectroscopy, and also in condense-matter physics of mesoscopic devices.

In order to demonstrate the significance of band-structure computation with a tight-binding model on dynamical properties of electrons in graphene, we first study Coulomb diagonal-dephasing (CDD) rate for induced optical polarization of thermally-excited electrons and holes around the Dirac point in an intrinsic (or undoped) graphene sample. For undoped graphene, conduction electrons can be introduced by a photo-excitation process [8], giving rise to equal number of electrons and holes $n_{e}=n_{h}\equiv n_{0}$, where $n_{0}$ represents the areal density of photo-excited carriers. For non-equilibrium photo-carriers under a transverse optical field, its induced optical coherence in steady states decays [1] with the sum of CDD rates $\Delta_{e}\left(k\right)$ and $\Delta_{h}\left(k\right)$ for electrons (e) and holes (h), respectively. These two rates determine the inhomogeneous line-shape of a resonant interband-absorption peak at $\hbar \omega =\varepsilon_{k}^{e}+\varepsilon_{k}^{h}$ for vertical transitions of electrons with their kinetic energies $\varepsilon_{k}^{e,h}$ in valence and conduction bands.

**Figure 7:**
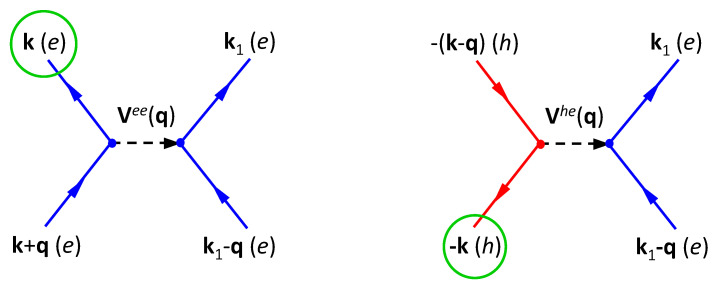
Feynman diagrams for CDD rate $\Delta_{e}\left(k\right)$ of electrons in Equation (34). (**left**) Coulomb coupling between pair of electrons in one inelastic-scattering event; (**right**) Coulomb coupling between an electron and a hole in another inelastic-scattering event.

As illustrated by Feynman diagrams [3] in Figure 7, the CDD rate $\Delta_{e}\left(k\right)$ of electrons is calculated as [1,44]

(34) $$\begin{array}{ccc} \Delta_{e}\left(k\right) & = & \frac{8\pi }{\hbar A^{2}}\;\sum_{k_{1},q\neq 0}{\left|V_{k,k_{1};\;k_{1}-q,k+q}^{\mathrm{ee}}\right|}^{2}\left[L_{0}\left(\varepsilon_{k_{1}-q}^{e}+\varepsilon_{k+q}^{e}-\varepsilon_{k_{1}}^{e}-\varepsilon_{k}^{e},\Gamma_{e}\right)\right.\\ & \times & \left.\left\{f_{k_{1}-q}^{e}\;f_{k+q}^{e}\;\left(1-f_{k_{1}}^{e}\right)+\left(1-f_{k_{1}-q}^{e}\right)\;\left(1-f_{k+q}^{e}\right)\;f_{k_{1}}^{e}\right\}\right]\\ & + & \frac{8\pi }{\hbar A^{2}}\;\sum_{k_{1},q\neq 0}{\left|V_{k,k_{1};\;k_{1}-q,k-q}^{\mathrm{he}}\right|}^{2}\left[L_{0}\left(\varepsilon_{k_{1}-q}^{e}+\varepsilon_{-(k-q)}^{h}-\varepsilon_{k_{1}}^{e}-\varepsilon_{-k}^{h},\Gamma_{\mathrm{eh}}\right)\right.\\ & \times & \left.\left\{f_{k_{1}-q}^{e}\;f_{-(k-q)}^{h}\;\left(1-f_{k_{1}}^{e}\right)+\left(1-f_{k_{1}-q}^{e}\right)\;\left(1-f_{-(k-q)}^{h}\right)\;f_{k_{1}}^{e}\right\}\right]\;, \end{array}$$

where both spin and valley degeneracies are included, A represents the surface area of graphene sample, the first and second terms correspond to the left and right panels of Figure 7, and both scattering-in and scattering-out contributions [44] are taken into consideration in these two terms. Moreover, $\varepsilon_{k}^{e,h}$ in Equation (34) stands for the kinetic energy of electrons (e) or holes (h), and $f_{k}^{e,h}={\left\{1+\exp\left[\left(\varepsilon_{k}^{e,h}-\mu_{e,h}\right)/k_{B}T\right]\right\}}^{-1}$ is the Fermi function for thermal-equilibrium photo-carriers with their chemical potentials $\mu_{e,h}$ at temperature *T*. Here, $\mu_{e,h}$ are separately determined by following two equations for given *T*, i.e.,

(35) $$n_{e,h}=\frac{4}{A}\;\sum_{k}\;\frac{1}{1+\exp[\left(\varepsilon_{k}^{e,h}-\mu_{e,h}\right)/k_{B}T]}\;,$$

where both spin and valley degeneracies are included and $\mu_{e}=\mu_{h}$ in our case. Furthermore, in Equation (34), $L_{0}\left(a,b\right)=\left(b/\pi \right)/\left(a^{2}+b^{2}\right)$ is the Lorentzian line-shape function, $\Gamma_{e,h}$ are inverse lifetime of unperturbed electrons or holes, and $\Gamma_{\mathrm{eh}}=\left(\Gamma_{e}+\Gamma_{h}\right)/2$.

In addition, we have introduced in Equation (34), as well as in Equation (40) below, the Coulomb-interaction matrix elements, given by [56]

(36) $$\begin{array}{ccc} V_{k,k_{1};\;k_{1}-q,k+q}^{\mathrm{ee}} & = & u_{c}\left(q\right)\;F_{k,\;k+q}^{\left(c\right)}\left(q\right)\;F_{k_{1},\;k_{1}-q}^{\left(c\right)}\left(-q\right)\;,\\ V_{k,k_{1};\;k_{1}-q,k-q}^{\mathrm{he}} & = & u_{c}\left(q\right)\;F_{k,\;k-q}^{\left(v\right)}\left(q\right)\;F_{k_{1},\;k_{1}-q}^{\left(c\right)}\left(-q\right)\;,\\ V_{k,k_{1};\;k_{1}-q,k+q}^{\mathrm{hh}} & = & u_{c}\left(q\right)\;F_{k,\;k+q}^{\left(v\right)}\left(q\right)\;F_{k_{1},\;k_{1}-q}^{\left(v\right)}\left(-q\right)\;,\\ V_{k,k_{1};\;k_{1}-q,k-q}^{\mathrm{eh}} & = & u_{c}\left(q\right)\;F_{k,\;k-q}^{\left(c\right)}\left(q\right)\;F_{k_{1},\;k_{1}-q}^{\left(v\right)}\left(-q\right)\;, \end{array}$$

where $u_{c}\left(q\right)=e^{2}/\left[2\epsilon_{0}\epsilon_{r}\;\left(q+q_{0}\right)\right])$ in Equation (36) is the two-dimensional Fourier transformed Coulomb potential ∼1/r including static screening, $\epsilon_{0}$ represents the vacuum permittivity, and $\epsilon_{r}=2.4$ is the average dielectric constant of the host material. Additionally, $q_{0}$ stands for the inverse Thomas-Fermi screening length, and can be given by a semi-classical model as [57]

(37) $$q_{0}=\left(\frac{e^{2}}{8\epsilon_{0}\epsilon_{r}k_{B}T}\right)\;\frac{4}{A}\;\sum_{k}\;\left[\cosh^{-2}\left(\frac{\varepsilon_{k}^{e}-\mu_{e}}{2k_{B}T}\right)+\cosh^{-2}\left(\frac{\varepsilon_{k}^{h}-\mu_{h}}{2k_{B}T}\right)\right]\;,$$

where both spin and valley degeneracies have been included.

Furthermore, the introduced $F_{k,\;k^{\prime }}^{\left(s\right)}\left(q\right)$ in Equation (36) with s=c,v represents the Bloch-function form factor, calculated as [57]

(38) $$\begin{array}{ccc} & & F_{k,\;k^{\prime }}^{\left(s\right)}\left(q\right)=\int d^{2}r\;{\left[\Phi_{k}^{\left(s\right)}\left(r\right)\right]}^{*}\;\exp\left(iq\cdot r\right)\;\Phi_{k^{\prime }}^{\left(s\right)}\left(r\right)=\frac{1}{N_{c}}\;\left\{{\left[a_{k}^{\left(s\right)}\right]}^{†}⊗a_{k^{\prime }}^{\left(s\right)}\right\}\;\\ & \times & \sum_{j,j^{\prime }=1}^{N_{c}}\;\exp\left[-i\left(q-k\right)\cdot \Delta_{j}-ik^{\prime }\cdot \Delta_{j^{\prime }}\right]\;\sum_{\ell ,\ell^{\prime }\in A,B}\;\exp\left[i\left(k^{\prime }-k+q\right)\cdot R_{\ell }\right]\;W_{s}\left(q,R_{\ell^{\prime }\ell }+\Delta_{jj^{\prime }}\right)\;,\;\;\;\; \end{array}$$

where the Bloch functions $\Phi_{k}^{c,v}\left(r\right)$ in Equations (5) and (22) have been employed. In Equation (38), $N_{c}$ represents the number of near-neighbor atoms within a unit cell, $\Delta_{j}$ stands for the lattice vectors of the near-neighbor atoms relative to the sublattice site $R_{\ell }$, and $a_{k}^{\left(s\right)}$ are two column eigenvectors in Equation (32) for s=c,v. The Wannier-function structure factor $W_{s}\left(q,R_{\ell^{\prime }\ell }+\Delta_{jj^{\prime }}\right)$ in Equation (38) is defined as

(39) $$W_{s}\left(q,R_{\ell^{\prime }\ell }+\Delta_{jj^{\prime }}\right)=\int d^{2}r\;{\left[\psi_{p_{z}}^{\left(s\right)}\left(r\right)\right]}^{*}\exp\left(iq\cdot r\right)\;\psi_{p_{z}}^{\left(s\right)}\left(r-R_{\ell^{\prime }\ell }-\Delta_{jj^{\prime }}\right)\;,$$

where $R_{\ell^{\prime }\ell }=R_{\ell^{\prime }}-R_{\ell }$ and $\Delta_{jj^{\prime }}=\Delta_{j}-\Delta_{j^{\prime }}$. In fact, Equations (34) and (36)–(39) are the key results in this paper for connecting the calculated tight-binding wave functions and band structures to a quantum-statistical theory for graphene optical properties.

**Figure 8:**
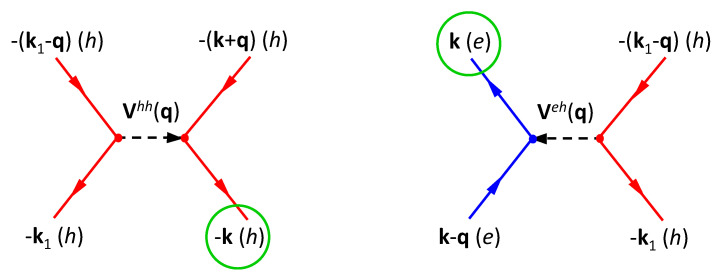
Feynman diagrams for CDD rate $\Delta_{h}\left(k\right)$ of holes in Equation (40). (**left**) Coulomb coupling between pair of holes in one inelastic-scattering event; (**right**) Coulomb coupling between a hole and an electron in another inelastic-scattering event.

Similarly, as illustrated by Feynman diagrams [3] in Figure 8, the CDD rate $\Delta_{h}\left(k\right)$ of holes takes the form [1,44]

(40) $$\begin{array}{ccc} \Delta_{h}\left(k\right) & = & \frac{8\pi }{\hbar A^{2}}\;\sum_{k_{1},q\neq 0}{\left|V_{k,k_{1};\;k_{1}-q,k+q}^{\mathrm{hh}}\right|}^{2}\left[L_{0}\left(\varepsilon_{-(k_{1}-q)}^{h}+\varepsilon_{-(k+q)}^{h}-\varepsilon_{-k_{1}}^{h}-\varepsilon_{-k}^{h},\Gamma_{h}\right)\right.\\ & \times & \left.\left\{f_{-(k_{1}-q)}^{h}\;f_{-(k+q)}^{h}\left(1-f_{-k_{1}}^{h}\right)+\left(1-f_{-(k_{1}-q)}^{h}\right)\;\left(1-f_{-(k+q)}^{h}\right)\;f_{-k_{1}}^{h}\right\}\right]\\ & + & \frac{8\pi }{\hbar A^{2}}\;\sum_{k_{1},q\neq 0}{\left|V_{k,k_{1};\;k_{1}-q,k-q}^{\mathrm{eh}}\right|}^{2}\left[L_{0}\left(\varepsilon_{k-q}^{e}+\varepsilon_{-(k_{1}-q)}^{h}-\varepsilon_{k}^{e}\;-\varepsilon_{-k_{1}}^{h},\Gamma_{\mathrm{eh}}\right)\right.\\ & \times & \left.\left\{f_{-(k_{1}-q)}^{h}\;f_{k-q}^{e}\;\left(1-f_{-k_{1}}^{h}\right)+\left(1-f_{-(k_{1}-q)}^{h}\right)\;\left(1-f_{k-q}^{e}\right)\;f_{-k_{1}}^{h}\right\}\right]\;. \end{array}$$

Computationally, the π-electron band structure of graphite can be obtained by employing the nearest-neighbor tight-binding model [58,59]. For graphene, the reciprocal lattice in the wave-vector space also acquires the hexagonal symmetry, same as that in real lattice. Moreover, the low energy bands are found linear and isotropic near the corners of the first Brillouin zone or *K* point. Such *K*-point linear bands become essential for the low-energy (or small wave-number) excitation of electrons. The calculated energy dispersions by diagonalizing the 2×2 Hamiltonian matrix are given by [58,59]

(41) $$\varepsilon_{k}^{e,h}=\pm \frac{3}{2}\;\gamma_{0}bk\equiv \hbar v_{F}k\;,$$

where $\gamma_{0}=2.4$ eV is the hopping integral between the nearest-neighbor atoms, b=1.42Å is the C–C bond length, and signs ± represents conduction (+) and hole (−) bands, respectively. Meanwhile, the corresponding spinor-type Bloch wave functions are found to be

(42) $$\phi_{p_{z},k}^{\left(c,v\right)}\left(r\right)=\frac{1}{\sqrt{2}}\;\left[U_{k}^{\left(1\right)}\left(r\right)\mp e^{i\theta_{k}}\;U_{k}^{\left(2\right)}\left(r\right)\right]\;,$$

where as shown in Equation (20), $U_{k}^{\left(1\right)}\left(r\right)$ and $U_{k}^{\left(2\right)}\left(r\right)$ are two sublattice Bloch functions built from the superposition of the periodic $2p_{z}$ orbitals, Ref. [59] and $\theta_{k}=\tan^{-1}\left(k_{y}/k_{x}\right)$ is the angle between the wave vector *k* and *x*-axis. As in Equation (22), we can further express the $2p_{z}$ atomic orbital by means of a generalized hydrogen-like wave function, given by [60]

(43) $$\psi_{p_{z}}\left(r\right)=C_{0}\;r\;\cos\theta \;e^{-Z^{*}r/2a_{0}}\;,$$

where $C_{0}$ is a normalization factor, $a_{0}$ the Bohr radius, and an effective nucleus charge number $Z^{*}$ is 3.18.

In particular, the structure factor introduced in Equation (38) can be calculated explicitly as

(44) $$\begin{array}{ccc} F_{k^{\prime },\;k}^{\left(s\right)}\left(q\right) & = & \delta_{k^{\prime },\;k+q}\;\int d^{2}r\;{\left[\Phi_{k+q}^{\left(s\right)}\left(r\right)\right]}^{*}\;\exp\left(iq\cdot r\right)\;\Phi_{k}^{\left(s\right)}\left(r\right)\\ & \approx & \delta_{k^{\prime },\;k+q}\;\langle \phi_{p_{z},\;k+q}^{\left(s\right)}\left(r\right)\;|\;\exp\left(iq\cdot r\right)\;|\;\phi_{p_{z},\;k}^{\left(s\right)}\left(r\right)\rangle \;, \end{array}$$

where s=c,v for Bloch wave function. Moreover, the Bloch-function structure factor in Equation (44) takes the form [60]

(45) $$\begin{array}{ccc} & & \langle \phi_{p_{z},\;k+q}^{\left(s\right)}\left(r\right)\;|\;\exp\left(iq\cdot r\right)\;|\;\phi_{p_{z},\;k}^{\left(s\right)}\left(r\right)\rangle \\ & = & \frac{1}{N_{A}+N_{B}}\;\sum_{R=R_{A},R_{B}}\;\langle \psi_{p_{z}}\left(r-R\right)\;|\;\exp\left[iq\cdot (r-R)\right]\;|\;\psi_{p_{z}}\left(r-R\right)\rangle \;\frac{1}{2}\left[1\pm \frac{\gamma \left(k+q\right)\;\gamma^{*}\left(k\right)}{\left|\gamma (k+q)\;\gamma (k)\right|}\right]\;,\;\;\;\;\;\;\;\;\;\;\;\; \end{array}$$

where tight-binding function $\psi_{p_{z}}\left(r\right)$ is given by Equation (43), and the signs (±) correspond to conduction (+) and valence (−) bands, respectively [59].

**Figure 9:**
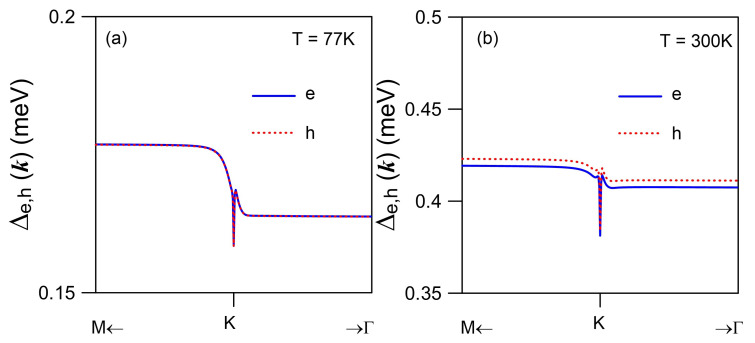
Calculated Coulomb diagonal-dephasing rates $\Delta_{e,h}\left(k\right)$ for electrons (e, blue solid curves) from Equation (34) and $\Delta_{h}\left(k\right)$ for holes (h, red dashed curves) from Equation (40) as functions of wave number *k* (with respect to k=0 at the *K* valley) at temperatures T=77K in (**a**) and T=300K in (**b**), where $\epsilon_{r}=2.4$ and $\Gamma_{e}=\Gamma_{h}=0.01\;$meV are assumed.

For intrinsic graphene, we have chemical potential $\mu_{e}=\mu_{h}=0$ [61]. However, there is still a finite intrinsic areal density $n_{i}\approx \left(\pi /6\right)\;{\left(k_{B}T/\hbar v_{F}\right)}^{2}$ due to thermal excitation of electrons and holes at finite temperatures *T*. In fact, we find $f_{k}^{e}=f_{k}^{h}=1/2$ at the *K* valley or k=0. Here, the calculated CDD rates from Equations (34) and (40), respectively, for electrons $\Delta_{e}\left(k\right)$ and holes $\Delta_{h}\left(k\right)$ are presented in Figure 9a at T=77 K and in Figure 9b at T=300K. Since $f_{k}^{e,h}∼\exp\left(-\varepsilon_{k}^{e,h}/k_{B}T\right)$ as $\varepsilon_{k}^{e,h}\gg k_{B}T$, the thermal occupations of electron and hole states will be limited mostly to wave numbers close to the *K* valley due to their lower kinetic energies $\varepsilon_{k}^{e,h}$ around k=0, as seen in Figure 6.

The Coulomb diagonal-dephasing rates $\Delta_{e,h}\left(k\right)$ presented in Figure 9a,b quantifies an amplitude-decay process of induced electron-hole optical coherence with wave vector ***k*** by an optical field towards the state before external perturbation. Furthermore, the Coulomb off-diagonal-dephasing rates $\Lambda_{e,h}\left(k,q\right)$ reveals deformations of induced optical-polarization waves with different wave vectors k+q [8].

Considering the fact that major occupations of electrons and holes are accumulated around k=0, we have $f_{k}^{e,h}\approx 0$ only if *k* is large. As a result, we find from Equation (34) that $f_{k_{1}-q}^{e}\;f_{k+q}^{e}\;\left(1-f_{k_{1}}^{e}\right)\ll 1$ at k=0 since we require $1-f_{k_{1}}^{e}\approx 1$ for large $k_{1}$, $f_{k+q}^{e}\approx 1$ for small *q*, and $f_{k_{1}-q}^{e}\approx 1$ for both large *q* and $k_{1}$, which, however, cannot be satisfied simultaneously. Similar conclusion can also be drawn for the second term in Equation (34), where we find $f_{k_{1}-q}^{e}\;f_{-(k-q)}^{h}\;\left(1-f_{k_{1}}^{e}\right)\ll 1$. Combining these two facts together, we expect that a dip will occur at k=0 for the Coulomb diagonal-dephasing rate $\Delta_{e}\left(k\right)$, as seen in Figure 9a. Moreover, the observed anisotropic energy dispersion in Figure 6a along the *K*-*M* and *K*-Γ directions directly leads to a staircase-like feature in Figure 9a for both $\Delta_{e}\left(k\right)$ and $\Delta_{h}\left(k\right)$. As temperature *T* is raised from 77K in Figure 9a to 300K in Figure 9b, the thermally-excited areal densities of electrons and holes are increased with $T^{2}$; therefore, the Coulomb interaction ($\propto T^{4}$) between electrons and holes, as well as the Coulomb interaction among electrons or holes, will be enhanced greatly. Consequently, we find that both $\Delta_{e}\left(k\right)$ and $\Delta_{h}\left(k\right)$ are enhanced by a factor of 2.4, in addition to amplified depth of the dip at k=0. Furthermore, different structural factors in Equation (39), corresponding to ± signs for conduction and valence bands, give rise to a slightly larger value of $\Delta_{h}\left(k\right)$ in comparison with that of $\Delta_{e}\left(k\right)$, as well as different dispersion features around the *K* valley for $\Delta_{e}\left(k\right)$ and $\Delta_{h}\left(k\right)$. These two computed Coulomb diagonal-dephasing rates can be physically applied to the spectral [32] and polarization [1,36] functions in order to study transport and optical properties of graphene material.

## 6. Carrier Energy-Relaxation Rate in Doped Graphene

In condensed-matter physics, the microscopic energy-relaxation time usually refers to a measure of the time it requires for one electron in the system to be significantly affected by the presence of other electrons, lattice vibrations, and randomly-distributed ionized impurity atoms in the system through an either scattering-in or scattering-out process mediated by electron-electron, electron-phonon and electron-impurity interactions, respectively. Since the microscopic energy-relaxation time is assigned to a specific electronic state, we are able to define a thermally-averaged energy-relaxation time through the diagonal density-matrix elements of electrons for all electronic states. In this way, one can reveal unique temperature dependence of this macroscopic energy-relaxation time and utilize it for simplifying the well-known Boltzmann transport equation within the relaxation-time approximation [44].

By going beyond the intrinsic graphene samples, we would like to investigate further the impurity scattering of electrons in extrinsic (or doped) graphene materials. In parallel with the discussion on scattering rates in Section 5, we present here the calculations for intraband-scattering of electrons by randomly-distributed impurities. Results for intraband-scattering of holes can be obtained in a similar way.

By using the detailed-balance condition, the microscopic energy-relaxation time $\tau_{\mathrm{rel}}\left(k\right)$ of electrons in the presence of randomly-distributed ionized impurities can be calculated according to [44]

(46) $$\frac{1}{\tau_{\mathrm{rel}}\left(k\right)}=W_{\mathrm{in}}\left(k\right)+W_{\mathrm{out}}\left(k\right)\;,$$

where the scattering-in rate for electrons in the final |k〉 state is

(47) $$\begin{array}{ccc} W_{\mathrm{in}}\left(k\right) & = & \frac{4\pi n_{\mathrm{im}}}{\hbar A}\;\sum_{q\neq 0}\;\left\{{\left|U_{k,\;k-q}^{\mathrm{im}}\left(q\right)\right|}^{2}f_{k-q}^{e}\;L_{0}\left(\varepsilon_{k}^{e}-\varepsilon_{k-q}^{e},\Gamma_{e}\right)\right.\\ & + & \left.{\left|U_{k,\;k+q}^{\mathrm{im}}\left(q\right)\right|}^{2}f_{k+q}^{e}\;L_{0}\left(\varepsilon_{k}^{e}-\varepsilon_{k+q}^{e},\Gamma_{e}\right)\right\}\;, \end{array}$$

whereas the scattering-out rate for electrons in the initial |k〉 state takes the form

(48) $$\begin{array}{ccc} W_{\mathrm{out}}\left(k\right) & = & \frac{4\pi n_{\mathrm{im}}}{\hbar A}\;\sum_{q\neq 0}\;\left\{{\left|U_{k+q,\;k}^{\mathrm{im}}\left(q\right)\right|}^{2}\left(1-f_{k+q}^{e}\right)\;L_{0}\left(\varepsilon_{k+q}^{e}-\varepsilon_{k}^{e},\Gamma_{e}\right)\right.\\ & + & \left.{\left|U_{k-q,\;k}^{\mathrm{im}}\left(q\right)\right|}^{2}\left(1-f_{k-q}^{e}\right)\;L_{0}\left(\varepsilon_{k-q}^{e}-\varepsilon_{k}^{e},\Gamma_{e}\right)\right\}\;. \end{array}$$

Here, $n_{\mathrm{im}}$ represents the areal density of ionized impurity atoms in the crystal, and $|U_{k,\;k^{\prime }}^{\mathrm{im}}{\left(q\right)|}^{2}$ comes from the randomly-impurity scattering of electron in the second-order Born approximation [48,62]. Explicitly, the random impurity-interaction matrix elements are calculated as

(49) $${\left|U_{k,\;k^{\prime }}^{\mathrm{im}}\left(q\right)\right|}^{2}=Z^{*2}{\left|u_{c}\left(q\right)\right|}^{2}\;{\left|\int d^{2}r\;{\left[\Phi_{k}^{\left(c\right)}\left(r\right)\right]}^{*}\;\exp\left(iq\cdot r\right)\;\Phi_{k^{\prime }}^{\left(c\right)}\left(r\right)\right|}^{2}=Z^{*2}{\left|u_{c}\left(q\right)\right|}^{2}\;{\left|F_{k,\;k^{\prime }}^{\left(c\right)}\left(q\right)\right|}^{2}\;,$$

where $Z^{*}$ is the charge number of ionized impurity atoms.

Substituting Equation (49) back into Equation (47), we obtain

(50) $$\begin{array}{ccc} W_{k}^{\mathrm{in}} & = & \frac{4\pi n_{\mathrm{im}}Z^{*2}}{\hbar A}\;\sum_{q\neq 0}\left\{f_{k-q}^{e}\;L_{0}\left(\varepsilon_{k}^{e}-\varepsilon_{k-q}^{e},\Gamma_{e}\right)\;{\left|u_{c}\left(q\right)\right|}^{2}\;{\left|F_{k,\;k-q}^{\left(c\right)}\left(q\right)\right|}^{2}\right.\\ & + & \left.f_{k+q}^{e}\;L_{0}\left(\varepsilon_{k}^{e}-\varepsilon_{k+q}^{e},\Gamma_{e}\right)\;{\left|u_{c}\left(q\right)\right|}^{2}\;{\left|F_{k,\;k+q}^{\left(c\right)}\left(q\right)\right|}^{2}\right\}\;, \end{array}$$

(51) $$\begin{array}{ccc} W_{k}^{\mathrm{out}} & = & \frac{4\pi n_{\mathrm{im}}Z^{*2}}{\hbar A}\;\sum_{q\neq 0}\left\{\left(1-f_{k+q}^{e}\right)\;L_{0}\left(\varepsilon_{k+q}^{e}-\varepsilon_{k}^{e},\Gamma_{e}\right)\;{\left|u_{c}\left(q\right)\right|}^{2}\;{\left|F_{k+q,\;k}^{\left(c\right)}\left(q\right)\right|}^{2}\right.\\ & + & \left.\left(1-f_{k-q}^{e}\right)\;L_{0}\left(\varepsilon_{k-q}^{e}-\varepsilon_{k}^{e},\Gamma_{e}\right)\;{\left|u_{c}\left(q\right)\right|}^{2}\;{\left|F_{k-q,\;k}^{\left(c\right)}\left(q\right)\right|}^{2}\right\}\;. \end{array}$$

Using the inverse microscopic energy-relaxation time in Equation (46), we can further calculate the macroscopic thermally-averaged energy-relaxation time $\tau_{\mathrm{rel}}\left(T\right)$ as a function of temperature *T*, yielding [44]

(52) $$\frac{1}{\tau_{\mathrm{rel}}\left(T\right)}=\frac{4}{n_{e}A}\sum_{k}\;\left[\frac{1}{\tau_{\mathrm{rel}}\left(k\right)}\right]f_{k}^{e}\;.$$

Actually, the results in Equation (46) and in Equations (50)–(52) demonstrate the approach for relating the computed tight-binding wave functions and band structures to graphene transport properties described by a many-body scattering theory. This calculated relaxation time in Equation (52) can be employed for building up different orders of moment equations [63] based on semi-classical Boltzmann transport equation [6] under the relaxation-time approximation [44]. Here, the zeroth-order moment equation [63] grantees the conservation of conduction electrons and allows us to find the chemical potential of electrons, as in Equation (35), for given areal doping density and temperature. Moreover, the first-order moment equation [63] makes it possible to find transport mobility and conductivity [64] for bias-field driven conduction electrons.

For doped graphene, we have Fermi energy $E_{F}=\hbar v_{F}\sqrt{\pi n_{0}}$ at low temperatures, Ref. [61] where $n_{0}$ represents the areal electron density from doping, i.e., $n_{0}=n_{\mathrm{im}}$ for completely ionized doping atoms. For low temperatures with $k_{B}T\ll E_{F}$, we have $f_{k}^{e}=\Theta \left(E_{F}-\varepsilon_{k}^{e}\right)$ or $f_{k}^{e}=\Theta \left(k_{F}-k\right)$, where Θ(x) is a unity step function and $k_{F}=\sqrt{\pi n_{0}}$ is the Fermi wave number.

**Figure 10:**
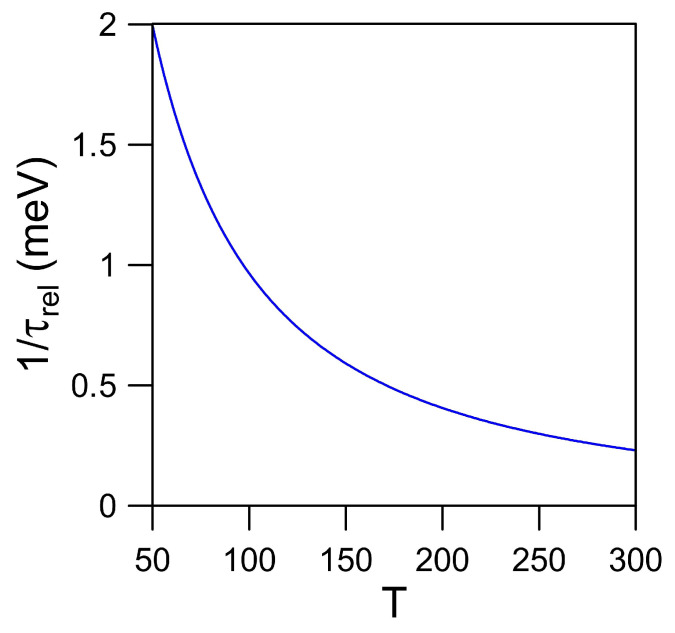
Calculated average energy-relaxation rate $1/\tau_{\mathrm{rel}}\left(T\right)$ from Equation (52) as a function of temperature *T* due to elastic scattering of doped electrons with impurities in graphene material, where $\epsilon_{r}=2.4$, $\Gamma_{e}=\Gamma_{h}=0.01\;$meV, $Z^{*}=1$, doped electron areal density $n_{0}=1\times 10^{11}\;$cm${}^{-2}$, and impurity areal density $n_{\mathrm{im}}=n_{0}$ are assumed.

Physically, the Coulomb diagonal-dephasing rates $\Gamma \left(k\right)=\Delta_{e}\left(k\right)+\Delta_{h}\left(k\right)$ in Figure 9 describes a decay process of induced electron-hole optical coherence, which is induced by an optical field over time, towards the state before perturbation. On the other hand, the electron energy-relaxation rate $1/\tau_{\mathrm{rel}}\left(T\right)$, determined by Equations (46) and (52), reflects the time, which is a quantum-statistical average over all occupied states of electrons, needed for recovering from a non-equilibrium-state occupation after an external perturbation to an initial thermal-equilibrium-state occupation before external perturbation via an elastic electron-impurity scattering process. Therefore, these two rates, as shown by Figure 9 and Figure 10, respectively, represent two fundamentally different microscopic physics mechanisms.

As seen from Figure 10, we find the electron energy-relaxation rate $1/\tau_{\mathrm{rel}}\left(T\right)$ reduces with increasing temperature *T* due to enhanced screening effect on Coulomb interaction $u_{c}\left(q\right)$ between two electrons or the rising of $q_{0}$ in Equation (37) with *T*, which implies that we have to wait a longer time $\tau_{\mathrm{rel}}\left(T\right)$ for our system returning to its initial thermal-equilibrium state at an elevated temperature. Furthermore, using the second-order Boltzmann moment equation [44], we would emphasize that this average energy-relaxation time $\tau_{\mathrm{rel}}\left(T\right)$, as determined from Equations (46) and (52), is directly associated with the mobility of transport electrons limited by elastic scattering from existence of impurities in the system.

## 7. Conclusions and Remarks

In conclusion, by introducing a generalized first-principles quantum-kinetic model coupled self-consistently with Maxwell and Boltzmann transport equations, we demonstrate the importance to incorporate inputs from first-principles band-structure computations for accurately describing non-equilibrium optical and transport properties of electrons in graphene. Generally speaking, the physical properties of an active material in a device are determined by both underlined band structures of involved materials and non-equilibrium responses to various external impulses.

In this study, we initialize with the tight-binding model for investigating band structures of solid covalent crystals by means of localized Wannier orbital functions, and further parameterize the hopping integrals in the tight-binding model for different covalent bonds. After that, we apply the general tight-binding-model formalism to graphene in order to acquire both band structures and wave functions of electrons within the whole first Brillouin zone of two-dimensional materials. For illustrating their significance, we utilize them to explore the intrinsic electron-hole Coulomb diagonal-dephasing rates used for spectral and polarization functions of graphene materials, and meanwhile, the energy-relaxation rate from extrinsic elastic scattering by impurities for transport mobility of doped electrons in graphene.

Theoretically, our current theory is capable of first-principles calculations of ultra-fast dynamics for non-thermal photo-generated electron-hole pairs. Simultaneously, this a theory also enables to describe electromagnetic, optical and electrical properties of semiconductor materials all together, as well as their interplay. Technologically, in combination with first-principles band-structure computations, the numerical output of current first-principles dynamics model can be used as an input for material optical and transport properties and put into a next-step simulation software, such as COMSOL Multiphysics, for a target device. Consequently, device characteristics can be predicted accurately for numerical bottom-up design and engineering.

## Figures and Tables

**Table 1:** Inter-atomic bonding parameters.

Bonding Parameter	Value [11]
$\eta_{s,s;\sigma }$	−1.40
$\eta_{s,d;\sigma }$	−3.16
$\eta_{d,d;\sigma }$	−16.2
$\eta_{s,p;\sigma }$	1.84
$\eta_{p,d;\sigma }$	−2.95
$\eta_{d,d;\pi }$	8.75
$\eta_{p,p;\sigma }$	3.24
$\eta_{p,d;\pi }$	1.36
$\eta_{d,d;\delta }$	0
$\eta_{p,p;\pi }$	−0.81

**Table 2:** Expressions for Bonding Integrals.

Bonding Integral	Expression [14]
$t_{s,s}$	$V_{s,s;\sigma }$
$t_{s,x}$	$\ell \;V_{s,p;\sigma }$
$t_{x,x}$	$\ell^{2}\;V_{p,p;\sigma }+\left(1-\ell^{2}\right)\;V_{p,p;\pi }$
$t_{x,y}$	$\ell m\;(V_{p,p;\sigma }-V_{p,p;\pi })$
$t_{x,z}$	$\ell n\;(V_{p,p;\sigma }-V_{p,p;\pi })$
$t_{s,xy}$	$\sqrt{3}\ell m\;V_{s,d;\sigma }$
$t_{s,x^{2}-y^{2}}$	$\left(\sqrt{3}/2\right)\;\left(\ell^{2}-m^{2}\right)\;V_{s,d;\sigma }$
$t_{3z^{2}-r^{2}}$	$[\left(n^{2}-\left(\ell^{2}+m^{2}\right)/2\right]\;V_{s,d;\sigma }$
⋮	⋮

**Table 3:** Graphene structure and tight-binding model parameters.

Parameter	Value [55]
$a_{1}$	$\left(a/2\right)\;\left(3,\sqrt{3}\right)$
$a_{2}$	$\left(a/2\right)\;\left(3,-\sqrt{3}\right)$
$a_{3}$	$\left(a/2\right)\;\left(0,2\sqrt{3}\right)$
$\delta_{1}$	$\left(a/2\right)\;\left(1,\sqrt{3}\right)$
$\delta_{2}$	$\left(a/2\right)\;\left(1,-\sqrt{3}\right)$
$\delta_{3}$	−a(1,0)
***K***	$\left(2\pi /3\sqrt{3}a\right)\;\left(\sqrt{3},1\right)$
$K^{\prime }$	$\left(2\pi /3\sqrt{3}a\right)\;\left(\sqrt{3},-1\right)$
$s_{nn}$	0.106
$s_{nnn}$	0.001
$t_{nn}$	−2.78eV
$t_{nnn}$	−0.12eV
$\varepsilon_{2}+C_{p}$	−0.36eV

## Data Availability

The datasets generated during and/or analyzed during the current study are available from the corresponding author on reasonable request.
